# The complete genome sequence of *Melia azedarach* Linn. (Meliaceae): a multi-purpose pesticide species

**DOI:** 10.1080/23802359.2020.1815600

**Published:** 2022-06-20

**Authors:** Boyong Liao, Jing Tan, Wei Zhou, Yi Wang, Yongquan Li, Xiaoyang Chen

**Affiliations:** a College of Horticulture and Landscape Architecture, Zhongkai University of Agriculture and Engineering, Guangzhou, Guangdong, China; b College of Forestry and Landscape Architecture, Guangdong Key Laboratory for Innovative Development and Utilization of Forest Plant Germplasm, State Key Laboratory for Conservation and Utilization of Subtropical Agro-Bioresources, South China Agricultural University, Guangzhou, Guangdong, China

**Keywords:** *Melia azedarach*, Meliaceae, pesticide species, complete chloroplast genome, automated assembly

## Abstract

The plant genus *Melia* has two or four species in the modern world, and is a natural source of a traditional pesticide, Toosendanin. In this study, we report the complete chloroplast genome of *Melia azedarach*, assembled from whole-genome high-throughput sequencing data, as a resource for future studies on the taxonomy and evolution of *Melia*. The chloroplast genome was 160,393 bp in length, with a large single-copy region of 87,598 bp, a small single-copy region of 18,709 bp, separated by two inverted repeat regions of 27,043 bp each. It was predicted to contain a total of 133 genes, with an overall GC content of 37.37%. Phylogenetic analysis placed *M. azedarach* closest to *Azadirachta* sp. in Meliaceae.

*Melia azedarach* Linn., a type species for the genus *Melia* Linn. (Meliaceae), has been a multi-purpose pesticide species for many years in China (Chen 1997; Peng and David 2008; Liao et al. 2016; Sivaraj et al. 2018). Four varieties of this species are thought to exist, i.e. *M. azedarach* Linn. (distributed in tropical and subtropical areas of the eastern hemisphere), *M. toosendan* Siebold et Zucc. (distributed around Sichuan in China), *M. dubia* Cav. (distributed from South of China to India), and *Melia volkensii* Gürke (distributed in Kenya, Tanzania, and Ethiopia) differing in several leaflet and fruit characteristics (Chen 1997; Peng and David 2008; Hanaoka et al. 2012; Liao et al. 2016; Sivaraj et al. 2018). However, the populations of varieties of *M. dubia* Cav. and *M. toosendan* Siebold et Zucc. differ based on nuclear SRAP markers and few DNA barcoding raising controversies that suggest that the current taxonomic treatment may not hold (Liao et al. 2016; Sivaraj et al. 2018). Also, to our knowledge, the taxonomic position of *Melia* within the family Meliaceae has not been evaluated phylogenetically. Thus, in this study, the complete chloroplast genome sequence of *M. azedarach* was assembled as a resource for future studies on the taxonomy of *Melia*. We also constructed a phylogeny to confirm its relationship with other genera within the family Meliaceae.

Sequence data from a whole-genome Illumina paired-end sequencing effort of a *M. azedarach* Linn. individual (unpublished) sampled from Guangzhou, Guangdong, China (location 23.109819 N, 113.276537 E; voucher B. Liao 20191 deposited in the Zhongkai University of Agriculture and Engineering Herbarium, ZUAE), was used for the assembly of this chloroplast genome. Approximately 4 Gb of paired-end (150 bp) sequence data was randomly extracted from the total sequencing output, as input into GetOrganelle (Jin et al. 2020) to *de novo* assemble the chloroplast genome with the seed sequences which distinguish sequences from chloroplast, mitochondrial, and nuclear genome. Annotation of the chloroplast genome was performed using the Geseq online tool (Tillich et al. 2017) and Geneious ver. 10.1 (http://www.geneious.com, Kearse et al. 2012), then manually verified and corrected by comparison with sequences of *Azadirachta indica* (GenBank: NC_023792).

The obtained complete chloroplast genome sequence of *M. azedarach* Linn. (GenBank accession MT460410) was 1,60,393 bp in length, with a large single-copy region of 87,598 bp, a small single-copy region of 18,709 bp, separated by two inverted repeat regions of 27,043 bp each. It was predicted to contain 133 genes, including 88 protein-coding genes, 37 tRNA genes, and 8 rRNA genes. The overall GC content was 37.37%.

To investigate the relationship between *Melia* and other genera within the family Meliaceae, a phylogenetic tree was constructed. The chloroplast genome of *M. azedarach* was aligned with 16 other chloroplast genomes of Meliaceae (Muellner et al. 2003; Koenen et al. 2015) and *Citrus aurantiifolia* (Christm.) Swingle chloroplast genome (GenBank accession NC_024929) as an outgroup, using MAFFT ver. 7.307 (Katoh and Standley 2013). A maximum-likelihood tree (Figure 1) was then constructed with RAxML using nucleotide substitution model GTRGAMMA, default matrix setting (Dayhoff), and algorithm (hill climbing), with a bootstrap value of 1000 replicates (Stamatakis 2014). *Melia azedarach* appears to be phylogenetically closest to Azadirachta sp.

**Figure 1.**
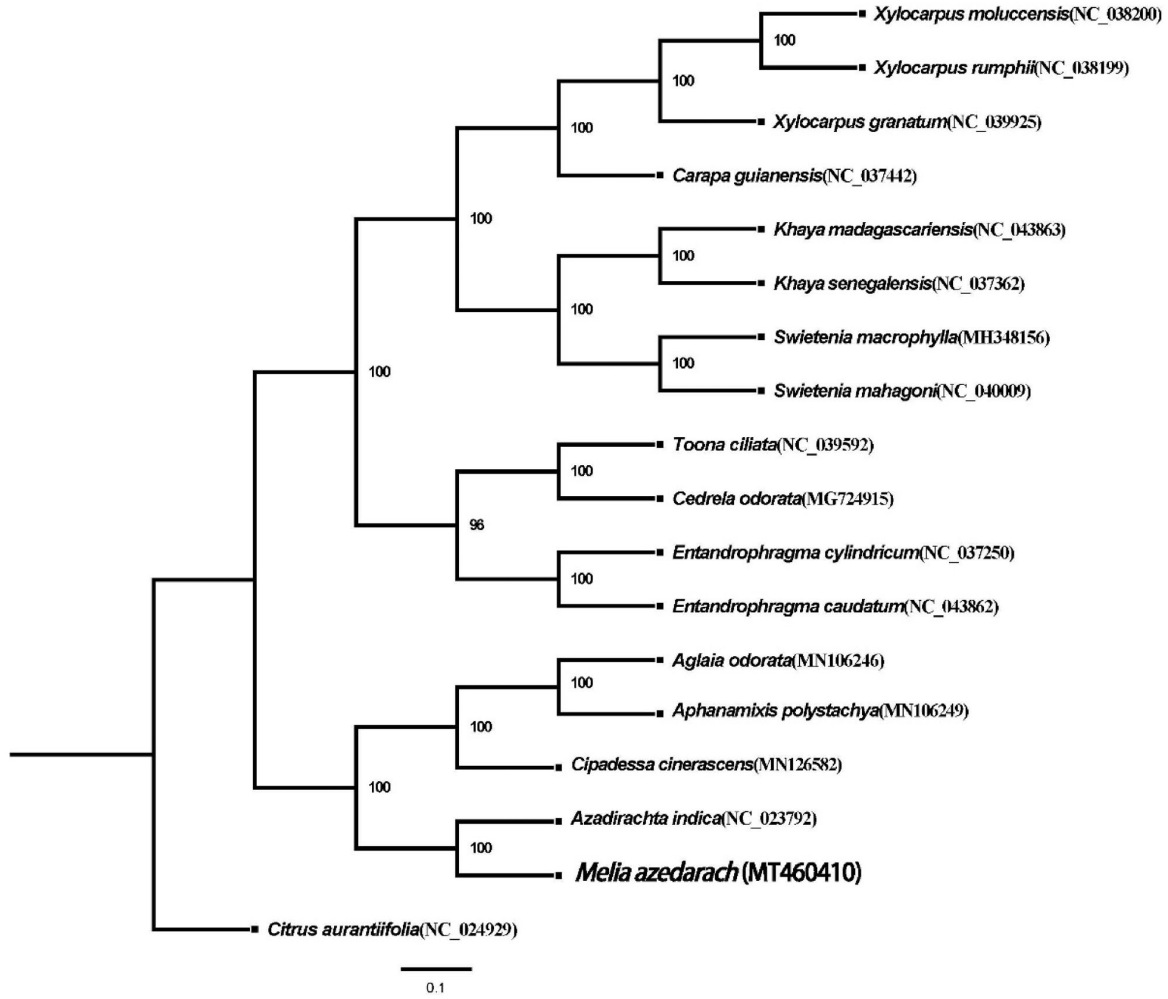
Maximum likelihood tree of Meliaceae based on complete chloroplast genomes, with *Citrus aurantiifolia* (Christm.) Swingle as outgroup. Bootstrap support values (based on 1000 replicates) are shown next to the nodes. Scale in substitutions per site.

## Data Availability

The data that support the findings of this study are openly available in National Center for Biotechnology Information at https://www.ncbi.nlm.nih.gov/, GenBank accession number is MT460410.
